# Subspecialty physicians’ perspectives on barriers and facilitators of hepatitis C treatment: a qualitative study

**DOI:** 10.1186/s12954-024-01057-z

**Published:** 2024-07-25

**Authors:** Erin Bredenberg, Catherine Callister, Ashley Dafoe, Brooke Dorsey Holliman, Sarah E. Rowan, Susan L. Calcaterra

**Affiliations:** 1 Division of Hospital Medicine, University of Colorado School of Medicine, 4th Floor, Leprino Building 12401 E 17th Ave, Aurora, CO 80045 USA; 2 Adult and Child Center for Health Outcomes Research and Delivery Science, Aurora, CO USA; 3 Denver Health and Hospital Authority, Denver, CO USA; 4 Division of Infectious Diseases, University of Colorado, Aurora, CO USA; 5 Division of General Internal Medicine, University of Colorado, Aurora, CO USA

**Keywords:** Hepatitis C, Substance-related disorders, Qualitative research

## Abstract

**Introduction:**

The hepatitis C virus (HCV) causes chronic and curable disease with a substantial burden of morbidity and mortality across the globe. In the United States (US) and other developed countries, incidence of HCV is increasing and people who inject drugs are disproportionately affected. However, HCV treatment rates amongst patients with substance use disorders (SUD) are suboptimal. In this study, we aimed to understand the perspectives of subspecialist physicians who care for substantial numbers of patients with HCV, including addiction medicine, infectious diseases, and hepatology physicians, to better understand barriers and facilitators of HCV treatment.

**Methods:**

We recruited subspecialty physicians via purposive and snowball sampling and conducted semi-structured interviews with 20 physicians at 12 institutions across the US. We used a mixed deductive and inductive approach to perform qualitative content analysis with a rapid matrix technique.

**Results:**

Three major themes emerged: (1) Perceptions of patient complexity; (2) Systemic barriers to care, and (3) Importance of multidisciplinary teams. Within these themes, we elicited subthemes on the effects of patient-level factors, provider-level factors, and insurance-based requirements.

**Conclusion:**

Our results suggest that additional strategies are needed to reach the “last mile” untreated patients for HCV care, including decentralization and leverage of telehealth-based interventions to integrate treatment within primary care clinics, SUD treatment facilities, and community harm reduction sites. Such programs are likely to be more successful when multidisciplinary teams including pharmacists and/or peer navigators are involved. However, burdensome regulatory requirements continue to hinder this expansion in care and should be eliminated.

**Supplementary Information:**

The online version contains supplementary material available at 10.1186/s12954-024-01057-z.

## **Introduction**

The hepatitis C virus (HCV) causes chronic, curable disease with a substantial burden of morbidity and mortality, affecting over 70 million people worldwide [1]. The introduction of well-tolerated and highly effective direct-acting antiretrovirals (DAAs) revolutionized HCV treatment and HCV-related mortality has decreased in the United States (US) since these medications came on the market in 2011 [2, 3]. HCV-related morbidity has decreased as well, with fewer HCV-associated hospitalizations and decreasing overall health care costs [4].

Despite these improvements in morbidity and mortality, the incidence of HCV has been increasing for over a decade [3]. The greatest increase occurred among people in younger age groups and corresponds to an increase in the use of non-medical opioids [3]. People who inject drugs (PWID) disproportionately bear the burden of HCV [5]. Current guidelines from national societies in the US, most notably the Infectious Diseases Society of America and the American Association for the Study of Liver Diseases (IDSA-AASLD), recommend treatment for all patients with HCV including those who are actively using substances [6]. Yet treatment uptake remains low in this population [7]. To meet goals set by the World Health Organization for eradication of HCV as a public health threat by the year 2030 [8], it is imperative to better understand the barriers to treating patients with HCV, particularly those with substance use disorders (SUD).

Prior qualitative work exploring the perspectives of patients with HCV has demonstrated that stigma, lack of education on HCV and its treatment options, and discomfort engaging with the healthcare system are barriers to HCV testing and treatment [9]. Medical providers have described barriers to HCV care along the continuum of care, including difficulty screening appropriate patients, hesitance to start patients on treatment due to concerns about adherence, and systemic challenges integrating HCV care within their practice [10–12]. Developing strategies to address barriers to treatment is especially critical in the wake of the COVID-19 pandemic, during which time screening, diagnosis, and treatment of HCV decreased [6]. In this qualitative study, we elicit the perspectives of subspecialty physicians to understand barriers and facilitators to the provision of HCV treatment. Our results will inform the development and implementation of interventions to increase provision of HCV treatment to patients with SUD.

## Materials and methods

### Study design

We conducted in-depth semi-structured interviews with subspecialty physicians in the fields of infectious diseases, hepatology, and addiction medicine. We limited interviews to these physician-types because their scope of practice often includes the care of patients with HCV. Three addiction medicine physicians (EB, CC, SC), with input from two qualitative experts (AD, BDH), designed the interview guide informed by the Practical, Robust Implementation and Sustainability Model (PRISM). PRISM contextual domains include Intervention, Recipients, Implementation & Sustainability Infrastructure, and External Environment (Appendix 1) [13]. We chose this framework to ensure that multilevel contextual factors affecting the provision of HCV treatment were considered. We also sought to understand subspecialty clinicians’ perspectives on a specific intervention, the initiation of HCV treatment during an acute care hospitalization; results of our findings regarding this intervention will be published separately. The interview guide was piloted with two addiction medicine physicians and results from the pilot interviews are included in our data set. This study was approved by the Colorado Multiple Institution Review Board.

### Participants and setting

To capture a range of experiences, we recruited physicians from across the US who provide care for patients with HCV, including clinicians with and without direct HCV treatment experience. We used a mix of purposive and snowball sampling to recruit our sample. We initially recruited participants using a purposive sampling approach [14] to include physicians from a variety of practice environments with varying levels of experience with HCV care. We emailed potential participants at local institutions to pilot the interview, including individuals affiliated with different institutions within the same city with a range of experience treating HCV in the hospital and outpatient setting. Next, we contacted potential participants at institutions across the country who had published or presented data at national conferences on this topic, as well as several additional individuals working at institutions that were geographically under-represented in our study. Lastly, at the conclusion of each interview, we used a snowball sampling technique, and asked participants for recommendations of potential participants with expertise or experience with HCV treatment. At the conclusion of each interview, participants were asked for contact information for others in the field (i.e., snowball sampling) [15]. Participants received a $50 gift card for participation.

### Data collection

Three interviewers trained in qualitative techniques (CC, EB, SC) conducted semi-structured interviews via remote videoconferencing between March and September 2023. Interviews were recorded, de-identified, and professionally transcribed. Interviews ranged from 27 to 77 min in length, with an average of 45 min. Interviewers completed an interview summary form after each interview and these forms were used to facilitate team discussion about the data and to assess for thematic saturation. Each interviewee also completed a short survey in REDCap following their interview. The survey contained questions on participant demographics and knowledge of, and comfort with, HCV treatment (Appendix 2).

### Analysis

We analyzed transcripts using a rapid matrix approach [16]. Rapid qualitative techniques may be more efficient than traditional qualitative analysis and have been demonstrated to achieve results that are as robust as traditional qualitative analysis when applied appropriately to a more straightforward research question [17, 18]. Initial deductive domains were derived from PRISM contextual domains and the interview guide. Then, these domains were piloted by three team members (EB, CC, AD) on three transcripts and additional inductive domains were added as needed. All transcripts were then reviewed and summarized into a matrix by two team members (EB, CC) using the finalized deductive and inductive domains as column headers. 20% of the transcripts were double-coded, reconciled, and summarized by the third team member (AD) to ensure fidelity of domain categorization. A content analysis of the matrix summaries was conducted through iterative team review and memoing of the data across interviews and domains to identify themes [19, 20]. Analysis was conducted across all participants and was not divided by subspecialist type or other demographic characteristics.

## Results

We interviewed 20 subspecialty physicians at 12 institutions across the US between March and September 2023. Ten addiction medicine, ten infectious diseases, and three hepatology physicians participated; three providers identified as subspecialists in both addiction medicine and infectious diseases (Table 1). Survey results show that most providers were comfortable with DAA treatment as evidenced by self-reported knowledge of how to identify patients who should be started on HCV treatment, self-reported knowledge of drug-drug interactions, and self-reported knowledge of which testing should be ordered prior to starting patients on HCV treatment (Table 1).

**Table 1:** Participant characteristics

*Participant characteristics* (*n = 20*^*a*^*)*	
Gender^b^	
Male	4 (26%)
Race	
White	14 (75%)
Black	0 (0%)
Asian	3 (16%)
More than one race/Other	2 (11%)
Ethnicity	
Hispanic	2 (11%)
Non-Hispanic	17 (89%)
Age (years)	
30–39	12 (63%)
40–49	6 (32%)
50–59	1 (5%)
Subspecialty provider type	
Addiction Medicine	10 (50%)^c^
Infectious Diseases (ID)	10 (50%)^c^
Hepatology	3 (15%)
U.S. geographic region	
Northeast	3 (15%)
Southeast	3 (15%)
Central	2 (10%)
West	12 (60%)
Comfort with DAA (Agree or Strongly Agree)	
Know how to identify appropriate patients to start on treatment	17 (89%)
Aware of important drug-drug interactions with DAA	16 (84%)
Know which tests should be order prior to starting DAA	16 (84%)

^a^One participant did not complete the quantitative survey

^b^All participants identified as cisgender

^c^Three participants self-reported dual-specialization in Addiction Medicine and Infectious Diseases

Three themes with four subthemes emerged. See Table 2 for a summary of themes with relevant recommendations to address the issues associated with each theme.

**Table 2:** Themes and recommendations

Theme	Relevant recommendations
Theme 1: Perceptions of patient complexity	A shift in strategy is needed to engage the “last mile” patients with HCV in the US
Subtheme 1a: Competing priorities	HCV testing and treatment should be low-barrier and integrated within primary care
	Non-medical professionals can help to link patients with HCV care through community-based organizations
	Telehealth-based services should be embedded within treatment for substance use disorders
Subtheme 1b: Variability in providers’ decisions to offer HCV treatment	Decentralization of HCV care can ensure equitable access for all patients
Theme 2: Systemic barriers to care	Public health departments should invest in infrastructure to support HCV testing and treatment in a variety of community-based settings
Subtheme 2a: Impact of insurance-based restrictions	Insurance-based restrictions for DAA must be eliminated, including: prior authorizations, specialty-specific restrictions on prescribing, limitations on treatment for people with substance use disorders, and requirements for medically unnecessary laboratory testing
Subtheme 2b: Impact of physician education	Education for medical providers who are not familiar with HCV care should emphasize the use of simplified algorithms for treatment and highlight reasons for referral to specialists
	Pharmacy-led protocols can support physicians who are less comfortable with HCV care
Theme 3: Importance of multidisciplinary teams	Effective teams to support patients through the HCV care continuum should include a pharmacist and a patient navigator
	Sustainable funding mechanisms are needed to support multidisciplinary HCV care teams and can be administered through departments of public health or other government entities

### Theme 1: perceptions of patient complexity

Participants noted that their patients with HCV represented a particularly complex patient population. Nearly all participants described common comorbidities of substance use and housing insecurity among their patients with HCV. Additionally, lack of reliable transportation and means for communication (e.g., cell phones) were often cited as specific barriers to ongoing engagement with HCV-related medical care.

In addition to social complexity, some physicians noted that co-occurring medical comorbidities could be barriers to HCV care. Hepatologists temporarily deferred HCV treatment for transplant candidates who could potentially receive an HCV-positive organ, or those with other, more pressing medical issues that could interfere with treatment.

Some physicians described a “last mile problem,” wherein the easiest patients to engage with HCV care have already been treated, leaving those who are most difficult to engage with care. They noted that engaging patients along the continuum of HCV care could be challenging.

*“Over the years*,* we have treated the vast majority of our easy-to-engage patients.” – Participant #14*,* ID*.

Some pointed out that engaging these “last mile” patients is especially important from a public health perspective, since patients with HCV and ongoing substance use are at particularly high risk of further transmission. They suggested the need for a shift in perspective when engaging these patients in HCV treatment, acknowledging that new strategies are needed to treat this population. Specific strategies for engagement included immediate initiation of HCV treatment upon diagnosis and decreasing the number of visits required for treatment.

### Subtheme 1a: competing priorities

Participants perceived that many patients with HCV had unstable, “chaotic” lives. This interfered with patients’ ability to engage with HCV care, as they often had more immediate unmet needs leading to lower prioritization of a chronic disease which generally does not have short-term medical consequences. Even when patients were interested in HCV treatment, physicians felt that these complex social issues led to challenges navigating the health care system and in turn, fewer HCV treatment starts and completions.

*“I have a lot of conversations with people where they say they’re really interested in [HCV treatment] but it’s not really their priority at that moment. Then there’s other people where they say it’s their priority*,* but then life is very chaotic when you’ve got a lot of other competing priorities and it’s very hard to get through that process.” – Participant #20*,* ID and Addiction*.

Clinicians discussed the need for low-barrier HCV care in the community to address these concerns.

### Subtheme 1b: variability in providers’ decisions to offer HCV treatment

Many participants expressed concerns about initiating HCV treatment for patients with barriers to follow-up due to concerns around the development of viral resistance if patients did not successfully complete a treatment course. Others described hesitancy to prescribe treatment for patients they perceived to be high risk for incomplete adherence to therapy because of concerns about insurance restrictions that would not allow a patient to be treated more than once with a costly course of DAA. Participants noted several reasons why patients may not complete a treatment course; many specifically cited concerns around housing insecurity impacting medication storage and concerns for medication theft or loss.

While most clinicians acknowledged these barriers, their decision of when to offer HCV treatment to patients with complex social situations varied. Several participants believed that clinicians should be more liberal about when to offer treatment, and not allow social comorbidities such as homelessness and substance use to be contraindications to offering DAA therapy. Some acknowledged that clinicians often cannot accurately identify which patients will successfully complete treatment and pointed out the public health benefits of engaging higher-risk patients in care.

*“I think it’s better to give people a chance than it is to say*,* ‘I don’t trust you to finish your meds*,* so I’m not gonna do it.’ People surprise me every time.” – Participant #19*,* Hepatology*.

Some clinicians worried that their own implicit biases might affect their decision to recommend treatment to patients. They acknowledged that offering treatment to patients they perceived as most likely to complete treatment could lead to inequitable care.

*“Then figuring out*,* is your life stable enough for you to finish the course and get the labs and things like that. Of course*,* in us making that decision there’s all sorts of biases that are coming out as far as who we’re selecting and who we’re priming for success or not. That’s fraught with our own selection bias.” – Participant #1*,* Addiction*.

### Theme 2 systemic barriers to care

Physicians familiar with HCV treatment generally perceived treating HCV as “easy” and “straightforward”, particularly for medically uncomplicated patients who can be treated using an algorithm-based approach. Some pointed out that modern treatments in the DAA era are much simpler, more effective, and better tolerated than older treatment regimens. Many felt that all clinicians should be comfortable with providing HCV care given its relative simplicity, including primary care providers. They pointed out that restricting HCV care to subspecialists could create unnecessary barriers to patient access to treatment.

*“To be quite honest with you*,* I think it’s not that difficult to treat hepatitis C. I don’t think it requires a specialized provider for the most part.” – Participant #16*,* ID*.

A few participants noted that treating HCV benefited patients in ways beyond the medical outcomes of treatment. They perceived that HCV treatment could enhance the patient-provider relationship and promote engagement with medical care overall. These clinicians highlighted the benefits of a broader range of clinicians incorporating HCV care into their practice.

Despite the relative medical simplicity of DAA regimens, physicians described many barriers to HCV treatment due to systems and organizational-level factors. Insurance-based barriers, inadequate provider education, and lack of organizational support structures were often cited as obstacles to HCV treatment. Participants noted that these organizational factors could deter individual clinicians from offering treatment. They perceived a need for an infrastructure to support clinicians to navigate administrative and systemic barriers to HCV treatment.

*“Individual primary care providers are much less consistently treating hepatitis C*,* often because they are disconnected from a larger infrastructure that gives them a guided plan for screening*,* diagnosis*,* treatment*,* and also resources.” – Participant #13*,* ID*.

#### Subtheme 2a: impact of insurance-based restrictions

Many participants noted that insurance coverage affected timely access to treatment for HCV. Patients without insurance or with inadequate insurance coverage faced particular challenges to treatment. Some clinicians described being unable to start uninsured patients on treatment due to uncertainty around the process of applying for coverage. A few clinicians experienced with the process of treating uninsured individuals noted that while manufacturers’ coupons exist that can offset the costs of treatment for uninsured individuals, the process for accessing these programs is burdensome.

Participants also noted that even among those with adequate insurance coverage, insurance-based restrictions medication approvals created barriers to care. These requirements included the need for prior authorization, genotyping prior to treatment initiation which is no longer recommended by national guidelines, [6] restrictions limiting medication fills to specific specialty pharmacies, and restrictions on prescribing to certain subspecialty provider types. Clinicians described insurance requirements as being overly burdensome and frequently at odds with medical best practices. Furthermore, differences in requirements between insurance companies regarding need for prior authorization, testing requirements, and preferred treatment regimens could be confusing and time-consuming.

*“Maybe if there weren’t the insurance barriers to the hepatitis C treatments*,* where we didn’t need the prior authorizations at the same level … you might see more hepatitis C treatment being done in addiction clinics.” – Participant #7*,* Addiction*.

Some participants noted that insurance barriers for DAAs had decreased over time, making prescribing easier. One provider who worked within the Veteran’s Affairs health care system noted that early coverage of DAAs was a major facilitator of treatment. A few perceived that further elimination of insurance requirements could lead to increased access to HCV treatment.

#### Subtheme 2b: impact of physician education

Interviewees’ own experience with treatment of HCV varied widely and many interviewees discussed inadequate provider education as a barrier to HCV treatment. Physicians who were less comfortable with DAAs described changing practice patterns, a lack of formal training, and inadequate bandwidth to actively seek out and maintain clinical knowledge on medication therapies for HCV as contributing to this gap.

*“Newer medications have come out*,* and it just was not on my radar as something that I personally would be prescribing because it seemed to be really kind of cohorted to GI*,* or ID physicians … I had so much to learn about and stay on top of anyway that I left it to those specialists to learn about and deal with.” – Participant #2*,* Addiction*.

Some physicians hypothesized that due to decreasing numbers of patients with HCV, sub-specialist trainees are encountering an insufficient volume of patients with HCV during training to become comfortable with independent management of the disease. Similarly, other interviewees described “sub-sub-specialization” in which a small number of subspecialty providers take ownership over a few specific disease processes as contributing to gaps in formal education for trainees.

Those with formal training in HCV care sometimes encountered specific practice patterns during fellowship that influenced their perceptions toward HCV treatment. One interviewee described receiving inaccurate information about HCV treatment recommendations in the setting of ongoing substance use during fellowship.

### Theme 3: importance of multidisciplinary teams

Many interviewees felt that having a multidisciplinary care team was helpful to manage the administrative burdens of HCV treatment. Clinicians viewed specialty pharmacists familiar with HCV treatment as particularly integral members of an HCV care team. The specific role of pharmacists varied by institution, but in nearly all cases they assisted with navigation of insurance issues including obtaining prior authorization when required. Pharmacists also variably assisted with medication selection and patient follow-up.

*“At our clinic the appointments are so short. There’s not enough time for me to see a patient a month in or if they have a side effect. I can’t work them in. Having the clinical pharmacist able to triage those questions*,* and see who really needs to be seen*,* and all that. She does pharmacist visits to do labs and review labs. That’s been really wonderful. It just offloads all the work that goes into treating hepatitis C.” – Participant #9*,* ID*.

Physicians noted that having a dedicated team member to assist with patient outreach and follow-up could be very helpful to engage the “last mile” patients with significant social barriers to medical care in the outpatient setting. While this role was sometimes filled by pharmacists as noted above, often it was filled by a case manager, social worker, or peer navigator. In some cases, the role did not exist at the interviewee’s institution, but they felt it would be helpful to have someone in that role.

*“We have a lovely team that supports our hep C treatment. Within primary care we have a pharmacist and a navigator whose primary job is to track down patients who have untreated hepatitis C*,* make sure that they get the appropriate lab testing done.” – Participant #3*,* Addiction*.

A few interviewees described funding limitations as a barrier to maintaining multidisciplinary teams. Funding could be unreliable, leading to gaps in care for patients receiving HCV treatment.

## Discussion

One major takeaway from our study was the description of a “last mile” problem regarding treatment of patients with HCV in the US, in which current population-level strategies to cure HCV have been highly successful amongst all but the most difficult-to-reach patients. Epidemiologic data shows a shift in the population with HCV, with incidence increasing among younger patients and PWID [3]. In prior qualitative studies, patients with HCV described a lack of access to medical care and an avoidance of health care services due to fear of stigma as major barriers to treatment engagement [9]. This aligns with concerns that interviewees in our study expressed about low perceived patient engagement with the health care system, supporting the need for innovative strategies to promote patient engagement. Overall, strategies to promote decentralization and integration of low-barrier HCV care in the community are effective [21]. These efforts can take many forms. One approach is integration of HCV treatment in primary care settings [6, 21–23]. A 2020 meta-analysis demonstrated that outcomes for HCV in the DAA era are comparable between subspecialty physicians and primary care physicians [24]. Initiation of HCV treatment during an acute care hospitalization is another novel strategy to capture patients who may otherwise be less likely to engage with the outpatient health care system [25]. Targeted outreach to specific subgroups at high risk for HCV, such as patients with criminal justice involvement, including those who are incarcerated or on probation, can also be efficacious [26–28]. Outreach programs for patients experiencing homelessness, particularly those that deploy rapid testing and treatment strategies, can help to meet patients where they are and promote engagement along the continuum of HCV care [29]. Embedding HCV testing and treatment within SUD treatment services, including opioid treatment programs, intensive outpatient programs, residential treatment centers or other SUD centers, or community harm reduction services may reach patients who are not accessing medical care elsewhere [30–35]. Although such programs are effective, few patients engaged with SUD treatment are screened for HCV and only a small minority of SUD treatment facilities offer HCV treatment [36].

Interviewees described barriers to deployment of the strategies above. Addiction medicine providers, community-based health care workers, and generalists including family and internal medicine practitioners may not have formal training on DAAs, and even hepatologists and infectious disease physicians may encounter insufficient numbers of patients during training to feel comfortable independently managing HCV. Indeed, one prior study demonstrated that the majority of primary care physicians refer patients with HCV to subspecialists for treatment [37]. One potential solution is to ensure clinicians have easy access to an “expert” in the field, such as a physician or pharmacist with specialized knowledge about HCV treatment. Remote, synchronous or asynchronous educational modules help clinicians feel more comfortable with treatment [22, 38]; however, as participants in our study pointed out, they may not have the time or desire to actively seek out these educational opportunities. Centralized telehealth-based interventions could address this gap and have proven effective when embedded within opioid treatment programs specifically [39].

Physicians also acknowledged many administrative hurdles to incorporating HCV treatment into practice, a finding that is well-supported by prior qualitative research [10, 12]. Many interviewees supported collaborative multidisciplinary teams to assist both patients and medical providers with navigation of the health care system. While peer navigation programs can be effective [32, 40], funding restrictions may limit their scope. Expanded funding through public health departments to support the needs of patients with HCV and the providers who care for them could lead to increased integration of HCV care within existing community and primary care services.

Many physicians in our study expressed hesitancy about starting patients with HCV and unstable social situations on DAAs due to concerns around incomplete treatment and the development of viral resistance, which could potentially lead to unnecessary delays in HCV care. Some acknowledged that attempting to select patients for treatment based on their likelihood of completion could be problematic, leading to biases in care provision. Indeed, there are racial and socioeconomic disparities in DAA treatment [41], and one prior study of HIV care providers in Connecticut found that implicit bias plays a role in providers’ decision to treat HCV [11]. Physicians in our study who were part of a multidisciplinary team expressed appreciation for support from team members, which generally included a pharmacist and often a patient navigator. Physicians practicing solo might feel more comfortable treating HCV despite social complexities such as homelessness if robust support systems were available to address patients’ social determinants of health.

Some interviewees highlighted perceived benefits to providing HCV treatment that extend beyond the prevention of long-term complications associated with the virus. This is in line with prior research demonstrating that patients perceive immediate physical and mental health benefits to treatment [42] and providers derive professional satisfaction from treating HCV [10] Providers should also acknowledge the public health benefits of addressing the “last mile” patients who are at highest risk of transmission, even if incomplete adherence to therapy is higher among some in this group. To this end, the most recent 2023 guidelines on HCV care from the IDSA-AASLD highlight the need to prioritize treatment for patients, and specifically describe the importance of taking a “treatment-as-prevention” approach among PWID [6]. These guidelines summarize data that incomplete adherence to DAA therapy is common, but that short periods of non-adherence are unlikely to result in treatment failure, and propose specific guidelines for management of patients with incomplete adherence to therapy [6]. The guidelines also support a minimal monitoring approach for most patients with uncomplicated HCV, in which follow-up after treatment initiation is limited to a single, telehealth-based visit to assess medication tolerability, followed by a laboratory visit post-treatment to determine sustained viral response (SVR) [6].

As many interviewees pointed out, insurance requirements can be burdensome and are often at odds with medical guidelines. Interviewees perceived this as a limitation to expanding the pool of providers willing and able to prescribe DAAs. Fortunately, barriers for Medicaid providers are decreasing in the US [43, 44]. However, insurance-based restrictions, including requirements for prior authorization, restrictions on DAA prescribing to certain subspecialist types, requirements for medically unnecessary laboratory testing such as genotyping prior to approval, and “sobriety requirements” which necessitate patients are abstinent from substances for a period of time prior to treatment initiation remain [43]. Eliminating these barriers has the potential to expedite care for patients, promote treatment retention, and encourage more clinicians to offer HCV care.

### Limitations

We used purposive and snowball sampling to ensure inclusion of clinicians with a range of experience with treatment of HCV. Due to the nature of the recruitment process, participants overall were likely to have a greater than average interest in the subject matter; indeed, the survey results shown in Table 1 support this, which could bias their views on HCV treatment. We specifically designed the interview guide to address this issue by broadly eliciting feedback on both barriers and facilitators of treatment.

Additionally, few hepatologists participated in our study. This is likely in part due to most study authors being addiction medicine clinicians without a network to recruit hepatologists specifically, which was compounded by snowball sampling in which clinicians of one type tended to refer to clinicians of the same type. However, we did not expect to find, nor did we attempt to explore, differences between provider types in our analysis. Furthermore, the inclusion of physicians from institutions across the US ensured perspectives from those practicing across a range of regulatory and political environments.

## Conclusion

Our study adds to a body of literature characterizing the need for low-barrier, integrated HCV care. Physicians in our study described patients with HCV as socially and/or medically complex and discussed challenges to engage patients in HCV care. Barriers to care included cumbersome administrative processes, limited provider education and variable attitudes toward treatment, and a lack of infrastructure to support providers and patients. Multidisciplinary teams can facilitate treatment, and elimination of overly restrictive insurance requirements for DAA prescribing could also help to expand the pool of providers willing and able to treat HCV.

### Electronic supplementary material

Below is the link to the electronic supplementary material.

Supplementary Material 1

Supplementary Material 2

## Data Availability

Data including audio recordings and transcripts cannot be shared openly due to participant privacy concerns.
