# Supplementary material for: Subspecialty physicians’ perspectives on barriers and facilitators of hepatitis C treatment: a qualitative study
Source: Harm Reduct J. 2024 Jul 25;21:140. doi: 10.1186/s12954-024-01057-z (PMC11271208; doi:10.1186/s12954-024-01057-z)
Supplement: Supplementary file 1 — Supplementary Material 1 [file 12954_2024_1057_MOESM1_ESM.docx]

**Appendix 2**

Questions:

*Each question is ranked on a Likert scale from 1-5 unless otherwise indicated.*

*1, Completely disagree*

*2, Disagree*

*3, Neither agree nor disagree*

*4, Agree*

*5, Completely agree*

Abbreviations*:*

*DAA = Direct-acting antiviral (medications to treat hepatitis C)*

*HCV = hepatitis C virus*

Participant ID:

1) Please provide the date on which you participated in an interview (format D-M-Y)

Demographics:

1) I am a: (Physician, Advanced Practice Provider)

2) I work in the field of (select all that apply): (Infectious Diseases, Hepatology, Addiction Medicine)

3) I identify as: (Male, Female, Other, Prefer Not To Say)

4) I consider my race to be: (White, Black, Asian, Other, Prefer Not To Say)

5) I consider my ethnicity to be: (Hispanic, Non-Hispanic, Prefer Not To Say)

6) My age is: (18-29, 30-39, 40-49, 50+, Prefer Not To Say)

Provider Attitudes and Experience:

1) I personally have ever written a prescription for DAA to patients with HCV while they are hospitalized. (Yes/No)

2) I know how to identify which patients with HCV are appropriate to start on DAA.

3) I am aware of important drug-drug interactions with DAA.

4) I know what tests and/or imaging must be completed before starting a patient on DAA.

5) Initiating a DAA to patients with HCV during a hospital stay is within my scope as a provider.

6) Management of chronic HCV with a DAA should be deferred to outpatient providers.

Acceptability of Intervention Measure (AIM):

1) Starting DAA to treat HCV while patients are in the hospital meets my approval.

2) Starting DAA to treat HCV while patients are in the hospital is appealing to me.

3) I like the idea of starting DAA to treat HCV while patients are in the hospital.

4) I welcome a process to start DAA to treat HCV while patients are in the hospital.

Intervention Appropriateness Measure (IAM):

1) Initiating DAA to treat HCV while patients are in the hospital seems fitting.

2) Initiating DAA to treat HCV while patients are in the hospital seems suitable.

3) Initiating DAA to treat HCV while patients are in the hospital seems applicable.

4) Initiating DAA to treat HCV while patients are in the hospital seems like a good match.

Feasibility of Intervention Measure (FIM):

1) Initiating DAA to treat HCV while patients are in the hospital seems implementable.

2) Initiating DAA to treat HCV while patients are in the hospital seems possible.

3) Initiating DAA to treat HCV while patients are in the hospital seems doable.

4) Initiating DAA to treat HCV while patients are in the hospital seems easy to do.
