# Supplementary material for: Subspecialty physicians’ perspectives on barriers and facilitators of hepatitis C treatment: a qualitative study
Source: Harm Reduct J. 2024 Jul 25;21:140. doi: 10.1186/s12954-024-01057-z (PMC11271208; doi:10.1186/s12954-024-01057-z)
Supplement: Supplementary file 2 — Supplementary Material 2 [file 12954_2024_1057_MOESM2_ESM.docx]

**Appendix 1**

*Interview guide is based on the PRISM framework, using the following domains: Intervention, Recipients, Implementation & Sustainability Infrastructure, and External Environment. In this context, Recipients includes both patients and providers.*

*Inclusion criteria: Participants must 1. Be subspecialty providers in the fields of Infectious Disease, Hepatology, or Addiction Medicine; 2. Currently work at least part of the time in an inpatient setting, and 3. Care for patients who have hepatitis C.*

*Goals: 1. To understand barriers and facilitators to the prescribing of medications to treat hepatitis C, 2. To understand provider attitudes toward initiating medications to treat hepatitis C during hospitalization, 3. To understand provider attitudes toward pharmacy-led interventions for treatment of hepatitis C*

Hello! My name is [name] and I am [role on the research team.] Thank you for agreeing to participate in our study. This interview should take between forty-five minutes and one hour of your time.

We at the University of Colorado Hospital are interested in learning more about providers’ experiences with treating hepatitis C. I will be asking you questions about your experience in both the outpatient and inpatient settings. Some questions may not apply to you and your work and that is OK. If a question does not apply, we will move on to the next question.

**Section 1: Provider Experience Treating HCV, Patients With HCV**

1. Tell me about your work with patients with hepatitis C. *(Recipients-Providers)*
2. Tell me about the patients with hepatitis C that you care for. (If needed, prompt further about general demographics, common comorbidities, common social and environmental factors) *(Recipients-Patients)*
3. Do you personally prescribe medications to treat HCV? *(Intervention, Recipients-Providers)*
   1. If YES –
      1. Tell me about prescribing these medications. *(Intervention)*
      2. What makes prescribing these medications easy? (*Implementation & Sustainability Infrastructure)*
      3. What makes prescribing these medications difficult? (*Implementation & Sustainability Infrastructure)*
      4. What would make it easier for you to prescribe medications to treat HCV? (*Implementation & Sustainability Infrastructure)*
      5. When do you hesitate to prescribe these medications? (*Implementation & Sustainability Infrastructure, External Environment)*
   2. If NO –
      1. Tell me more about that. (*Implementation & Sustainability Infrastructure)*
      2. What makes prescribing these medications difficult? (*Implementation & Sustainability Infrastructure)*
      3. What would make it easier for you to prescribe medications to treat HCV? (*Implementation & Sustainability Infrastructure)*
4. Tell me about a specific patient you saw who was treated for HCV, either in the outpatient or the inpatient setting. (You may not know the answer to some of these follow-up questions, and that is OK). *(Recipients-Patients)*
   1. Tell me more about the process for treating HCV. *(Intervention)*
   2. Who prescribed treatment for the patient’s HCV*? (Intervention)*
   3. What went well about the process? *(Implementation & Sustainability Infrastructure)*
   4. What did not go well about the process? *(Implementation & Sustainability Infrastructure)*
5. Tell me about a specific patient you saw who had untreated HCV. *(Recipients-Patients)*
   1. What got in the way of treatment? *(Recipients-Patients, External Environment)*
   2. What could have made treating the patient’s HCV easier? *(Recipients-Patients, External Environment)*
   3. What non-medical supports may have benefited this patient, if available? (Probe for social needs such as housing resources, SUD treatment, insurance coverage) *(External Environment)*
6. In an ideal world, how do you think the patient’s HCV be treated? *(Intervention)*
   1. Who should ideally be responsible for treating the patient’s HCV? *(Recipients-Providers)*
   2. When and where would the patient ideally start treatment? *(Intervention)*
   3. What would follow-up look like for this patient? *(Intervention, Implementation & Sustainability Architecture)*
   4. What barriers exist to this process in the real world? (Probe for institutional, insurance, policy barriers) *(Implementation & Sustainability Infrastructure, External Environment)*

**Section 2: Interventions to Increase Treatment of HCV**

Part A: Now I am going to show you a process map for prescribing medications to treat HCV among currently hospitalized patients which we are currently using at our institution. I am going to ask you more about your thoughts about this process and how it might be implemented at your own institution:

1. What are your initial thoughts about this process? *(Intervention)*
2. Tell me about this process at your institution, if one exists. (If they say the process doesn’t exist, skip to b). *(Intervention)*
   1. If the process exists:
      1. Tell me about a patient who was treated at your institution using this process. *(Recipients-Patients)*
      2. What about the process at your institution work well? *(Intervention, Intervention & Sustainability Infrastructure)*
      3. What barriers do you face? (Probe for patient, provider, institutional, and policy-level barriers). *(Intervention, Intervention & Sustainability Infrastructure, External Environment)*
      4. What are your thoughts on your process as compared to the one outlined above? What aspects of each process might work better than the other? What aspects of each process might be more difficult? *(Intervention)*
      5. Were you involved in the creation of this process? If YES – ask the questions below: *(Intervention)*
         1. What made implementing this process easy?  *(Intervention, Implementation & Sustainability Infrastructure)*
         2. What made implementing this process difficult? (Probe for patient, provider, institutional, and policy-level barriers) *(Intervention, Implementation & Sustainability Infrastructure, External Environment)*
   2. If the process doesn’t exist
      1. What do you think about a process like this at your institution? (Probe for patient, provider, and institution-level benefits) *(Intervention, Recipients-Patients, Recipients-Providers)*
      2. What might get in the way of implementing this process at your institution? (Probe for patient, provider, institutional-level, and policy-level barriers) *(Recipients-Patients, Recipients-Providers, Implementation & Sustainability Infrastructure, External Environment)*
      3. What might make this process easy to implement at your institution?  *(Implementation & Sustainability Infrastructure, External Environment)*
      4. What would motivate you to implement this process? *(Intervention)*
      5. What would make this process sustainable at your institution? *(Implementation & Sustainability Infrastructure, External Environment)*
3. Do you have any final thoughts about this process?

Part B: Finally, I am going to ask you some questions about pharmacist-led interventions for the treatment of HCV. In this model, clinical pharmacists may identify gaps in treatment and work with physician partners to prescribe key medications, or directly prescribe medications to treat certain disease states. For example, pharmacists often help to manage anticoagulation (specifically warfarin).

1. Tell me about an experience with a pharmacy-led intervention.
   1. What went will about this process?
   2. What could have gone better in this process?
2. What do you think about pharmacist-led interventions for the treatment of HCV? (Probe for potential benefits and drawbacks).

Final thoughts:

1. Anything that you thought we would ask but didn’t during this interview?
2. Do you have any recommendations for other people we could contact to participate in this study?

Wrap-up:

Thank you for your time! As soon as we conclude this interview, I am going to send you an anonymous survey with some questions about your demographic information and also attitudes toward prescribing of medications to treat hepatitis C among hospitalized patients. Please take the remaining 10 minutes of this hour to complete the survey. We will email you a $50 Amazon gift card within 1 week of completing the survey.
